# Liver Iron Retention Estimated from Utilization of Oral and Intravenous Radioiron in Various Anemias and Hemochromatosis in Humans

**DOI:** 10.3390/ijms21031077

**Published:** 2020-02-06

**Authors:** Annelies J. van Vuren, Richard van Wijk, Eduard J. van Beers, Joannes J.M. Marx

**Affiliations:** 1 Van Creveldkliniek, University Medical Center Utrecht, Utrecht University, 3584 CX Utrecht, The Netherlands; 2 Department of Clinical Chemistry and Haematology, University Medical Center Utrecht, Utrecht University, 3584 CX Utrecht, The Netherlands; 3 Departments of Haematology and Internal Medicine, University Medical Center Utrecht, Utrecht University, 3584 CX Utrecht, The Netherlands

**Keywords:** hemochromatosis 1, iron 2, NTBI 3, hereditary anemia 4, iron deficiency 5

## Abstract

Patients with hereditary hemochromatosis and non-transfusion-dependent hereditary anemia develop predominantly liver iron-overload. We present a unique method allowing quantification of liver iron retention in humans during first-pass of ^59^Fe-labeled iron through the portal system, using standard ferrokinetic techniques measuring red cell iron uptake after oral and intravenous ^59^Fe administration. We present data from patients with iron deficiency (ID; N = 47), hereditary hemochromatosis (HH; N = 121) and non-transfusion-dependent hereditary anemia (HA; N = 40). Mean mucosal iron uptake and mucosal iron transfer (±SD) were elevated in patients with HH (59 ± 18%, 80 ± 15% respectively), HA (65 ± 17%, 74 ± 18%) and ID (84 ± 14%, 94 ± 6%) compared to healthy controls (43 ± 19%, 64 ± 18%) (*p* < 0.05) resulting in increased iron retention after 14 days compared to healthy controls in all groups (*p* < 0.01). The fraction of retained iron utilized for red cell production was 0.37 ± 0.17 in untreated HA, 0.55 ± 0.20 in untreated HH and 0.99 ± 0.22 in ID (*p* < 0.01). Interestingly, compared to red blood cell iron utilization after oral iron administration, red blood cell iron utilization was higher after injection of transferrin-bound iron in HA and HH. Liver iron retention was considerably higher in HH and HA compared to ID. We hypothesize that albumin serves as a scavenger of absorbed Fe(II) for delivering albumin-bound Fe(III) to hepatocytes.

## 1. Introduction

Iron absorption and iron utilization studies performed in the 1960s and 1970s from last century using radiolabeled iron greatly improved our understanding of iron metabolism [1,2,3]. Over the last 25 years advances in molecular biology and genetics revolutionarily increased the knowledge on normal and abnormal iron biology including the discovery of hepcidin as key player in primary and secondary hemochromatosis [4,5].

Both patients with hereditary hemochromatosis and with non-transfusion-dependent hereditary anemia develop predominantly hepatic iron-overload. They share a common etiology: very low levels of hepcidin, while hepcidin is increased in anemia of inflammation [4]. Production of red blood cells (RBCs) requires 20–25 mg iron daily under physiologic conditions. The demand is supplied by import of iron from enterocytes (1–2 mg), iron recycling by macrophages (20–25 mg) and iron from storage tissues like hepatocytes [6]. Due to the absence of a renal or hepatic iron excretion mechanism, body iron levels are only controlled by intestinal absorption. The tendency to retain iron exposes humans to a substantial risk of iron-overload and related toxicity [7].

The hepcidin/ferroportin axis regulates iron export from enterocytes, macrophages and iron storage tissue into the circulation. Hepcidin inhibits iron efflux by binding to ferroportin, which leads to degradation of the exporter [4,6,8,9,10]. Inversely, less hepcidin synthesis or activity will result in increased intestinal iron transfer from enterocytes to the circulation [11]. Duodenal enterocytes, having a life-span of only two days while moving from the crypts of Lieberkühn to tips of the villi, express the divalent metal transporter 1 (DMT1) at the luminal side and intracellular ferritin, incorporating excess iron, which is rapidly lost to the lumen of the gut with exfoliating enterocytes. High expression of ferroportin results in iron depletion of enterocytes, by diffusion of Fe(II) through the basolateral membrane to a compartment where Fe(II) is rapidly oxidized with binding of Fe(III) to a variety of ligands, including transferrin [6]. In patients with hereditary hemochromatosis genetic inactivation of one of the intermediaries of the hepcidin-activation route results in inhibition of bone morphogenetic protein(BMP)-SMAD signaling and, subsequently, reduced hepatic hepcidin expression [12]. The most common forms of hereditary hemochromatosis are caused by mutations of the *Hfe* (Hereditary FE), hemojuvelin (*Hjv*), hepcidin (*Hamp*) or transferrin receptor 2 (*Trf2*) gene. Hereditary hemolytic or dyserythropoietic anemias are characterized by an increased effective or ineffective erythropoietic response [13]. Erythropoietin is the main hormone controlling erythropoiesis. Erythroblasts secrete erythroferrone in response to erythropoietin [14]. Erythroferrone suppresses hepatic hepcidin expression via inhibition of BMP-SMAD signaling [15]. So, both patients with hereditary hemochromatosis and non-transfusion-dependent hereditary anemia are at risk for development of iron-overload in response to low hepcidin [16,17,18,19].

Iron transport via transferrin seems not to contribute significantly to continuous hepatic iron loading in hemochromatosis, because both transferrin and hepatic transferrin receptor 1 (TfR1) are downregulated under iron-overload conditions. Still, livers of iron-loaded mice take up more iron than non-iron-loaded livers [20,21,22]. The importance of a non-transferrin-dependent iron-regulating system was demonstrated in hpx (atransferrinemic) mice, characterized by anemia and mainly hepatic iron-overload. Iron taken up by hepatocytes of hpx mice has to be non-transferrin-bound iron (NTBI) [20]. Hence, hepatic import of iron reversibly bound to other carrier molecules significantly contributes to continuous hepatic iron-overloading.

Here, we present data of radiolabeled iron absorption and iron utilization studies performed in a large cohort of patients with iron deficiency, primary and secondary hemochromatosis. The combination of these studies in the same patient provides unique information on iron transport after entrance of iron from ferroportin into the portal venous system and its uptake into hepatocytes during the first passage through the liver.

## 2. Results

Our cohort contained 278 analyses of subjects with normal iron stores, patients with iron deficiency, and with primary or secondary iron-overload with and without iron reducing treatment. Analyses in the non-transfusion-dependent hereditary anemia group without treatment were obtained in patients diagnosed with congenital sideroblastic anemia (CSA, N = 13), hereditary spherocytosis (N = 3), congenital dyserythropoietic anemia (CDA, N = 5), non-transfusion dependent β–thalassemia (NTDT, N = 2), HbE/β–thalassemia (N = 1), pyruvate kinase deficiency (PKD, N = 1) and Hb Adana (N = 4). Analyses in non-transfusion-dependent hereditary anemia patients with iron reducing therapy were obtained in patients with hereditary spherocytosis (N = 3), hexokinase deficiency (HKD, N = 2) and congenital sideroblastic anemia (CSA, N = 6). Of the treated non-transfusion-dependent hereditary anemia patient group, three analyses were obtained in patients on iron chelation therapy (deferoxamine), and eight analyses in patients on a regular phlebotomy scheme. All patients with hereditary haemochromatosis (HH, N = 121) were regarded as homozygotes, based on HLA-typing, and clinical and family history. Ferritin assays were not yet available when normal subjects were investigated. All patients in hereditary hemochromatosis patient group treated with iron reducing therapies were on a regular phlebotomy scheme, and one patient was both phlebotomized and treated with deferoxamine. Patient characteristics and median laboratory parameters are summarized in the Table 1.

### 2.1. Body Iron Retention is Significantly Increased in patients with Non-Transfusion-Dependent Hereditary Anemia and Hereditary Hemochromatosis

Median percentages of mucosal iron uptake, mucosal iron transfer and iron retention are summarized in Table 1. Mean mucosal iron uptake, and mucosal transfer were significantly higher in hereditary hemochromatosis and non-transfusion-dependent hereditary anemia patients without iron reducing treatment, and in iron deficient patients, compared to healthy controls (*p* < 0.05). Together, this resulted in a significantly increased iron retention after 14 days in all groups, with or without iron reducing treatment, when compared to healthy subjects (*p* < 0.01).

### 2.2. Disappearance Half-Life of Transferrin-Bound is Shorter in Non-Transfusion-Dependent Hereditary Anemia than in Hereditary Hemochromatosis

**Figure 1:**
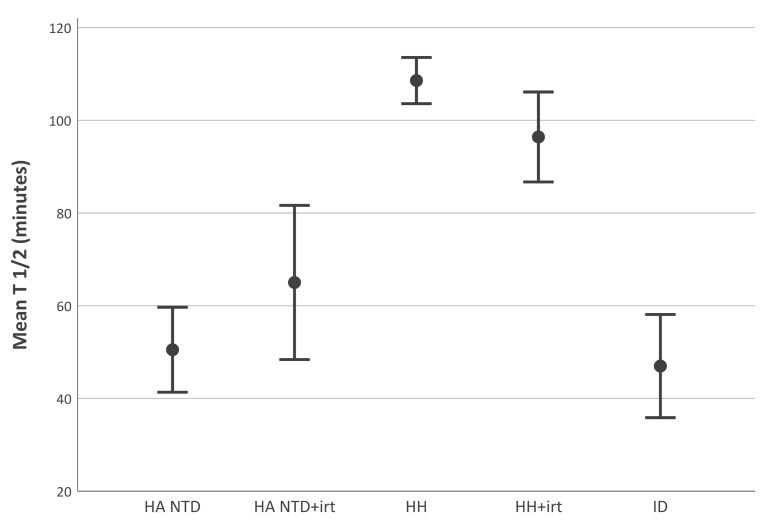
Disappearance half-life of transferrin-bound iron. Disappearance half-life of iron was calculated from blood samples taken at 0, 5, 15, 30, 45, 60, 90 and 120 min after injection. Half-life is expressed in minutes. Plot of the mean half-life categorized per disease group. Error bars represent 95% confidence intervals. HA NTD hereditary anemia, non-transfusion dependent; HH hereditary hemochromatosis; irt iron reducing therapy (phlebotomies or deferoxamine); ID iron deficiency; T½ half-life; min minutes.

We tested the velocity of disappearance of a single injection of transferrin-bound iron from the circulation. A graphic overview is provided in Figure 1. Mean disappearance half-life of transferrin-bound iron (± standard deviation) was significantly shorter in patients with iron deficiency (47 ± 37 min), untreated hereditary anemia (51 ± 23 min) and treated hereditary anemia (65 ± 22 min), than in treated (96 ± 29min) and untreated (108 ± 22 min) hereditary hemochromatosis patients (all *p* < 0.01). Half-life of iron in patients with non-transfusion-dependent hereditary anemia was comparable with iron deficient patients (*p* > 0.05). Clearance of radioiron from plasma in healthy controls was previously studied [23], and varied between 75–105 min (N = 6). Clearance in healthy controls is thereby much faster than in untreated patients with hereditary hemochromatosis, while those patients come close to the normal range during phlebotomy. Patients with hereditary anemia and effective erythropoiesis show a higher demand for iron by erythroblasts, due to hemolysis. These patients have a functional iron deficiency while iron due to early red cell destruction is accumulating in macrophages in spleen and liver.

Of interest was the data of three spherocytosis patients of whom ferrokinetic studies were available before and after splenectomy. We observed striking differences in plasma iron half-life before splenectomy (22, 15 and 23 min), and two years after splenectomy (70, 63 and 67 min respectively). Increase in spleen size was negatively correlated with half-life in the whole group of non-transfusion-dependent hereditary anemia patients (r = −0.50, 95% CI [−0.69; −0.26], *p* < 0.01). Hemolytic biomarkers were negatively correlated with half-life of iron: absolute reticulocyte count (r = −0.41, 95% CI [−0.51; −0.40], *p* = 0.02), serum lactate dehydrogenase (r = −0.49 [−0.66; −0.27], *p* < 0.01) and serum bilirubin (r = −0.53, 95% CI [−0.64; −0.42], *p* < 0.01). In all patients, half-life of iron was positively correlated with serum hemoglobin concentrations (r = 0.61, 95% CI [0.49, 0.72], *p* < 0.01). Notably, in congenital sideroblastic anemia, a disease characterized by disrupted utilization of iron in erythroblasts, ineffective erythropoiesis and relative low reticulocyte counts [24], half-life of iron was significantly longer than in other forms of non-transfusion-dependent hereditary anemia (63 ± 21 min versus 45 ± 23 min, 95% CI [4.5; 31.4], *p* = 0.02).

In summary, disappearance half-life of intravenous transferrin-bound iron was significantly longer in hereditary hemochromatosis patients compared to the patients with various anemias. In non-transfusion-dependent hereditary anemia patients more severe anemia and higher levels of hemolytic parameters were associated with shortened half-life of iron.

### 2.3. Red Blood Cell Iron Utilization Decreases When Iron-Overload Increases

**Figure 2:**
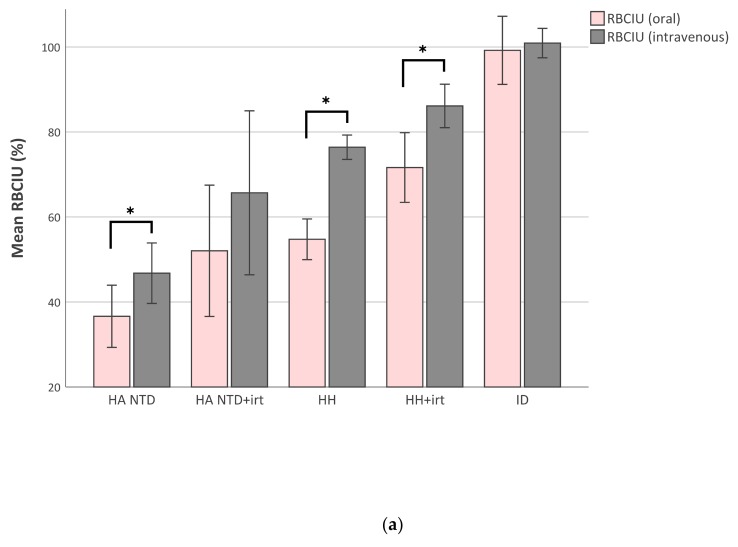

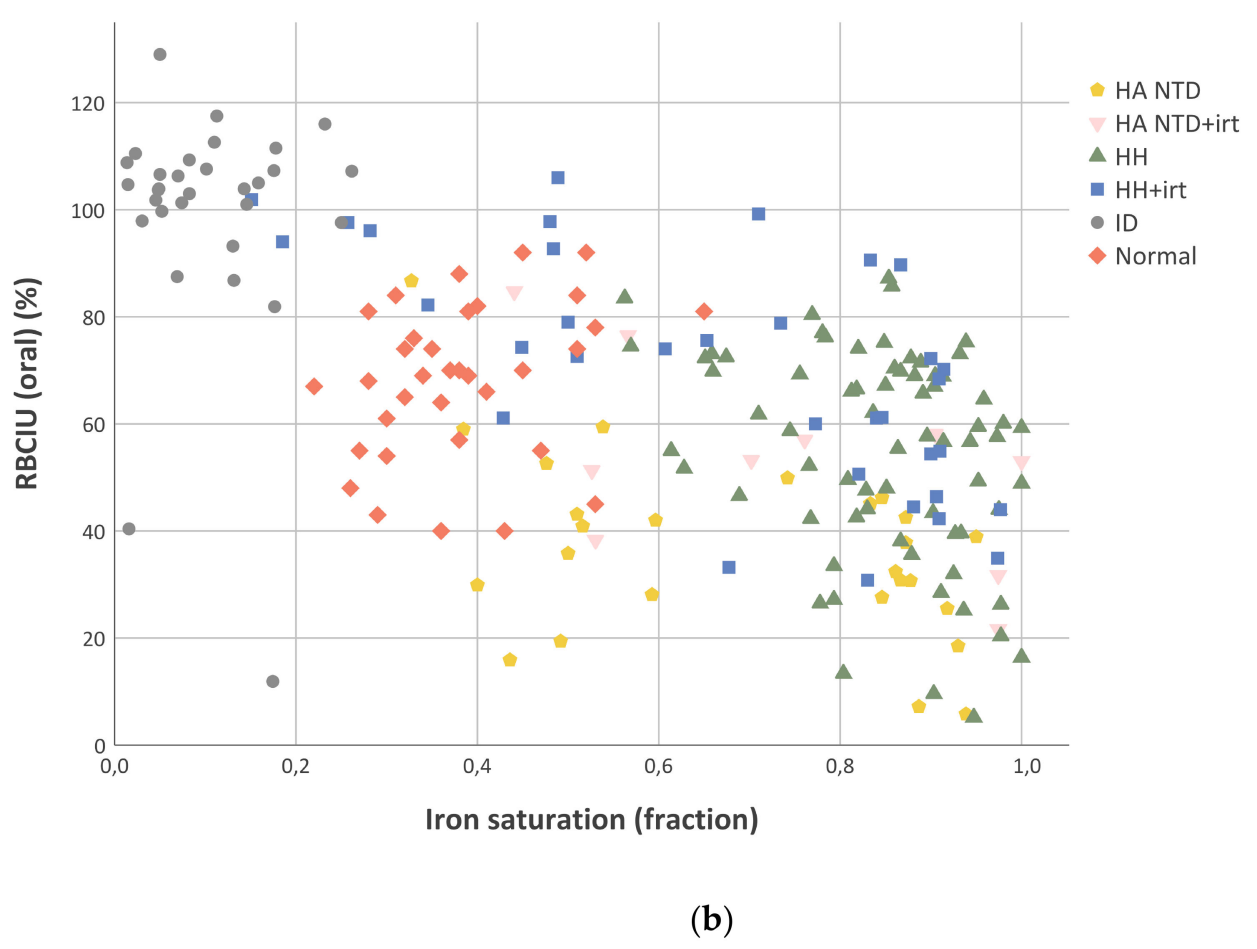
Red blood cell iron utilization (RBCIU). RBCIU was calculated from blood samples obtained 14 days after oral or intravenous iron test dose: RBCIU (oral) (%) = (^59^Fe in 1 mL blood × blood volume (mL))/^59^Fe retained in the body after oral iron dose × 100%, and RBCIU (intravenous) (%) = (^59^Fe in 1 mL blood × blood volume (mL))/^59^Fe iv dose × 100%. (**a**) Bar plot with mean percentages of RBCIU. Error bars represent 95% confidence intervals. *p < 0.01. (**b**) Scatter plot with iron saturation (x-axis) and RBCIU after oral iron (y-axis). Every dot represents a single analysis and is colored by diagnosis. HA NTD hereditary anemia non-transfusion dependent; HH hereditary hemochromatosis; ID iron deficiency; irt iron reducing therapy (phlebotomies or deferoxamine).

We measured the amount of ^59^Fe iron in peripheral blood samples 14 days after the iron test dose to determine the amount of iron utilized for RBC production. Mean percentages (± standard deviation) of RBC iron utilization (RBCIU) after an oral iron test dose were 37 ± 17% in untreated non-transfusion-dependent hereditary anemia, 53 ± 19% in treated non-transfusion-dependent hereditary anemia, 55 ± 20% in untreated hereditary hemochromatosis, 70 ± 22% in treated hereditary hemochromatosis, and 99 ± 22% in iron deficient patients. We previously reported, using the same methodology, a mean RBCIU of 82 ± 13% in ten healthy adults from an oral iron test dose [25]. In iron deficient patients the utilization of oral (99 ± 22%) and intravenous (100 ± 9%) iron were comparable. Surprisingly, RBCIU was significantly higher after intravenous iron than after an oral test dose in patients with hereditary anemia (47 ± 18%) or hereditary hemochromatosis (76 ± 12%), both untreated (Figure 2A; *p* < 0.01). In healthy controls, reported previously [25], RBCIU of intravenous iron was 85 ± 17%, and thereby comparable with the RBCIU after an oral iron test dose.

To understand intergroup differences in iron utilization, we investigated the influence of the pre-existing iron load in all patients and of the degree of anemia and hemolysis in patients with non-transfusion-dependent hereditary anemia. Iron saturation was negatively correlated with RBCIU after oral iron (r = −0.62, 95% CI [−0.71; −0.51], *p* < 0.01) and after intravenous iron (r = −0.56, 95% CI [−0.65; −0.47], *p* < 0.01) (Figure 2B), as was ferritin (respectively r = −0.55, 95% CI [−0.63; −0.45], *p* < 0.01; and r = 0.42, 95% CI [−0.55; −0.32], *p* < 0.01). Thus, in patients with a higher iron saturation or ferritin value, representing more severe iron-overload, the amount of iron utilized for RBC production was lower. The treated and untreated hereditary anemia patients were further analyzed to investigate the existence of a correlation between RBCIU and disease severity, based on laboratory parameters of erythropoiesis and hemolysis. The hemoglobin concentration was significantly correlated with RBCIU of intravenous iron (r = 0.55, 95%CI [0.30; 0.75], *p* < 0.01) and of oral iron (r = 0.61, 95% CI [0.33; 0.78], *p* < 0.01). RBCIU was not related to reticulocyte count, lactate dehydrogenase or bilirubin. Again, we observed striking differences in the three spherocytosis patients of whom data was available before and after splenectomy. All three patients were anemic before splenectomy and hemoglobin concentrations normalized after splenectomy; patients were phlebotomized after splenectomy. Utilization of oral and intravenous iron for RBC production improved significantly after splenectomy (mean oral RBCIU before splenectomy 41 ± 10%, after splenectomy 73 ± 14%, delta 32%, *p* = 0.04; mean intravenous RBCIU before splenectomy 50 ± 6%, after splenectomy 85 ± 13%, delta 36%, *p* = 0.03). In all non-transfusion-dependent hereditary anemia patients increase in spleen size (centimeters under the costal margin) was correlated with lower intravenous RBCIU (r = −0.59, 95% CI [−0.73; −0.45], *p* < 0.01) and less oral RBCIU (r = -0.50, 95% CI [−0.70; −0.27], *p* < 0.01).

Summarizing, the amount of iron utilized for RBC production was lower in patients with primary or secondary hemochromatosis compared to iron deficient patients and healthy controls. In non-transfusion-dependent hereditary anemia patients, iron utilization for RBC production was even more suppressed in patients with lower hemoglobin concentrations and splenomegaly.

### 2.4. Liver Iron Retention is Increased in Iron-Overload

**Figure 3:**
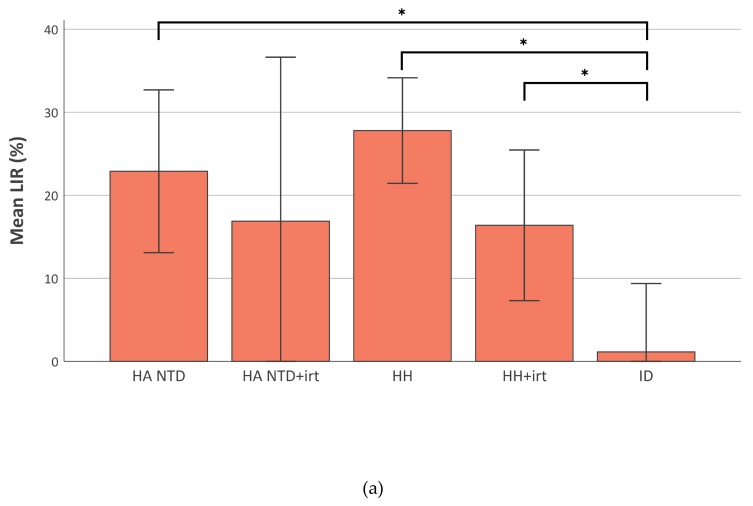

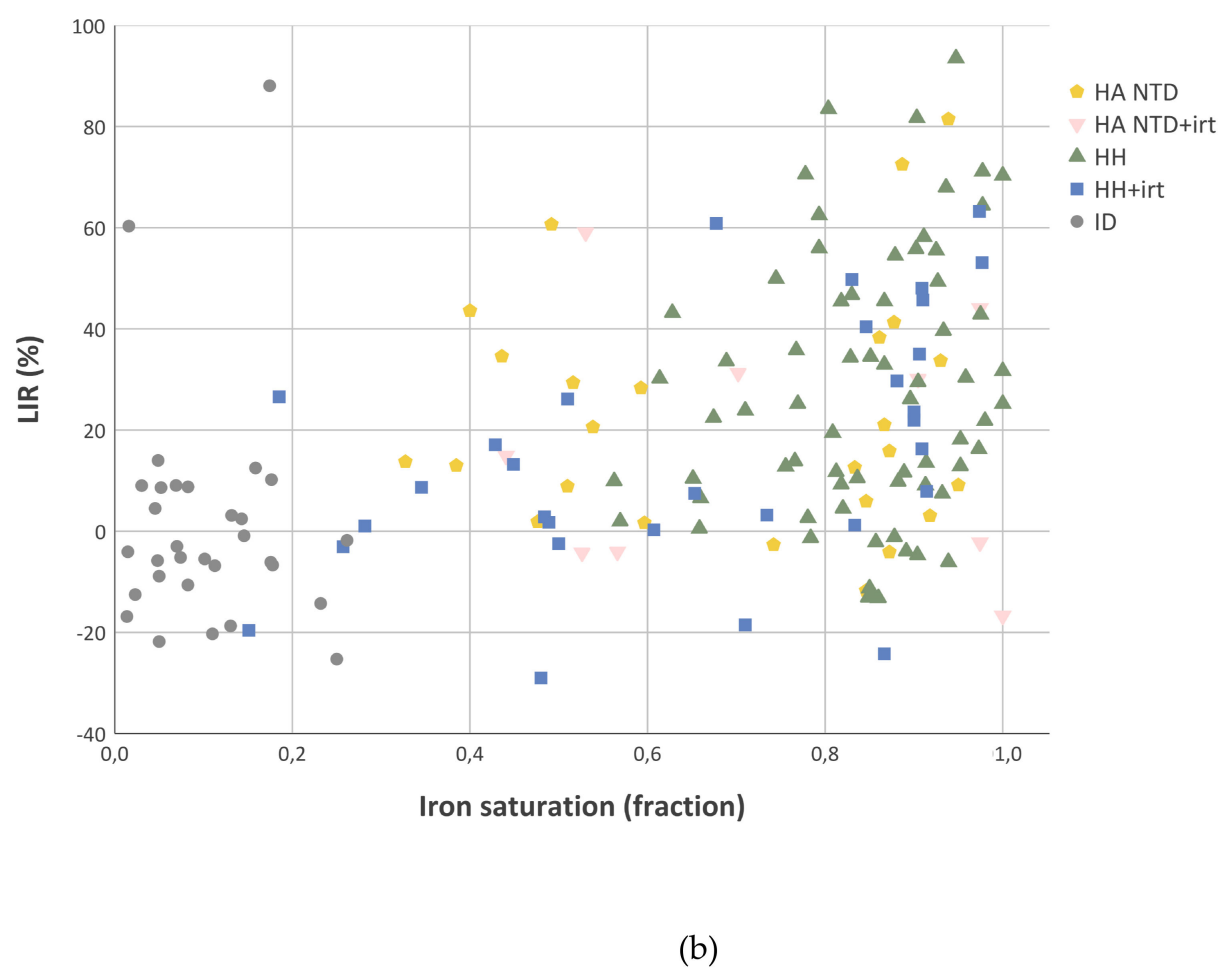
Liver iron retention (LIR). LIR calculated from the difference between red blood cell iron utilization (RBCIU) after intravenous and oral iron test dose and expressed as percentage of intravenous RBCIU: LIR (%) = (RBCIU intravenous–RBCIU oral)/RBCIU intravenous × 100%. (**a**) Bar plot with mean LIR values. Error bars represent 95% confidence intervals. *p < 0.05 (**b**) Scatter plot with iron saturation (x-axis) and LIR (y-axis). Every dot represents a single analysis and is colored by diagnosis. HA NTD hereditary anemia non-transfusion dependent; HH hereditary hemochromatosis; ID iron deficiency; irt iron reducing therapy (phlebotomies or deferoxamine).

We observed a difference between the utilization of oral and intravenous iron for RBC production in non-transfusion-dependent hereditary anemia and hereditary hemochromatosis patients (Figure 3A). The difference between oral and intravenous RBCIU was expressed as percentage of intravenous RBCIU and denominated as LIR (liver iron retention). Liver iron retention was close to zero in patients with iron deficiency anemia. Ferritin, serum iron and iron saturation fraction were very low. Free iron binding sites on transferrin could easily accommodate all iron entering the plasma after transport across the basolateral membrane of duodenal cells by ferroportin. The LIR had a mean value (± standard deviation) of 28 ± 26% in untreated hereditary hemochromatosis, 23 ± 24% in untreated hereditary anemia, 16 ± 25% in treated hereditary hemochromatosis patients, all significantly higher than the LIR of 1 ± 22% measured in iron deficient patients (*p* < 0.05) (Figure 3A). The LIR was strongly correlated to iron saturation (r = 0.41, 95% CI [0.26; 0.53], *p* < 0.01) (Figure 3B) and ferritin level (r = 0.47, 95% CI [0.30; 0.61], *p* < 0.01). Thus, the fraction of retained iron not utilized for erythropoiesis is considerably increased in patients with primary or secondary hemochromatosis.

## 3. Discussion

We report here on a unique combination of iron absorption and ferrokinetic data from a large cohort of patients with iron deficiency, primary hemochromatosis, hereditary anemia and secondary hemochromatosis, enabling quantification of liver iron retention.

An older study reported already in 1982 a significantly lower ^59^Fe-RBCIU in healthy aged subjects 14 days after an oral iron test dose, while RBCIU after an intravenous dose showed no difference [25]. In this small study RBCIU upon intravenous iron could not be measured in the same subjects as RBCIU upon oral iron administration. The authors suggested that the lower RBCIU after an oral dose could probably be a result of increased liver iron retention. In the present study we compared RBCIU after oral with RBCIU after intravenous iron administration in the same subject, demonstrating that both iron mucosal uptake (via DMT1) and mucosal transfer (via ferroportin) were increased in patients with iron deficiency anemia, primary and secondary hemochromatosis. In non-transfusion-dependent hereditary anemia and hereditary hemochromatosis patients a substantial part of absorbed iron was not utilized for erythropoiesis and the amount was highly dependent on the degree of iron-overload. Furthermore, we showed that half-life of transferrin-bound iron in patients with non-transfusion-dependent hereditary anemia is comparable with iron deficient patients but approximately half that in hereditary hemochromatosis patients.

The intravenous test dose of transferrin-bound ^59^Fe-labeled iron could rapidly interact directly with iron in the peripheral venous system. In our studies oral iron was administered as 7 mg ferrous ammonium sulphate (1 mg ^59^Fe(II)), which enters the portal vein via ferroportin. Part of this ^59^Fe will bind to apotransferrin in plasma, similar to the injected ^59^Fe, and will be cleared mainly by erythroblasts and other cells expressing transferrin receptors. The remaining ^59^Fe will bind to other molecules with formation of NTBI and labile plasma iron (LPI), resulting in iron deposition in the liver.

NTBI can be detected in patients with non-transfusion-dependent hereditary anemia and hereditary hemochromatosis with moderately increased transferrin saturation (TSAT) levels [26,27,28,29]. Short-lived amounts of NTBI can also be detected after supplemental ingestion of iron, even in patients with very low TSAT values [30,31]. Such values were obtained by venapuncture. Strikingly, we observed that in hereditary and secondary hemochromatosis the amount of retained iron of a ^59^Fe radiolabeled oral test dose of only 1 mg Fe(II) that was utilized for RBC production was lower when compared to the utilization of injected ^59^Fe radiolabeled transferrin-bound iron. This iron must have been deposited at a site with high iron storage capacity, most probably in hepatocytes.

We suggest that Fe(II) after entering the plasma in the portal circulation, after oxidation due to hephaestin in the basolateral membrane and plasma ceruloplasmin, will not only bind to free Fe(III) binding sites on transferrin but also to a variety of molecular species with affinity for iron (NTBI). In iron deficiency, with very low transferrin iron saturation, iron absorption will result in a negligible amount of NTBI, whereas in iron-overload, characterized by highly saturated plasma transferrin, iron absorption will result in significant amounts of NTBI in the portal circulation. This NTBI will be available as LPI [26,32]. LPI has a much lower affinity for Fe(III) than apotransferrin with a chemically labile character and a high propensity for redox reactions [32,33,34].

The amount of iron entering the portal vein after iron absorption is much smaller than that released from catabolized hemoglobin in macrophages of the spleen and the liver, also into the portal vein. Additional iron transport capacity will be needed for temporary storage of iron in hepatocytes. There are two candidates that may serve as scavengers of iron in the portal system in situations where not enough free iron-binding sites on transferrin are available: citrate and albumin [35]. The plasma concentration of citrate is maintained at about 100–120 μM. Albumin exists in the circulation at an extremely high concentration (35–50 g/L plasma). Albumin has a large number of negative carboxylate sites on its surface, suitable for binding of Fe(III). Albumin is able to bind iron in the presence and in the absence of citrate, and can bind Fe(III) even when transferrin is not fully saturated [36]. Albumin can be considered as a safe iron transporter in plasma, next to transferrin, that is able to donate iron to cells that do not have transferrin receptors, and is able to transport iron into hepatocytes.

In mice, ZIP14, member of the Zrt- and Irt-protein (ZIP) family of metal-ion transporters [35,37,38], efficiently transports NTBI into the intracellular compartment of hepatocytes [39,40]. In humans, ZIP14 is most abundantly expressed in the liver, and at lower levels in pancreas and heart [20,39]. Hepatic ZIP14 expression is upregulated in iron-loaded rats, which illustrates its regulation by iron itself [20].

**Figure 4:**
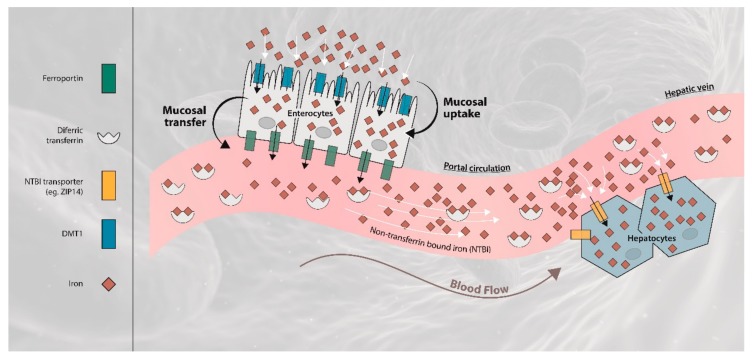
Continuous iron loading in primary or secondary hemochromatosis. Simplified visualization of iron absorption and first passage of the liver. Iron absorption in iron-overload results in generation of NTBI, including LPI. The majority of LPI produced in the portal system is taken up in the liver in the first-pass. DMT1 divalent metal transporter 1; NTBI non-transferrin bound iron.

Iron accumulation in hepatocytes was observed in a murine model for (juvenile) hemochromatosis resulting from *Hjv* knockout or *Hfe2* knockout with intact ZIP14 expression. However, mice with *Hfe* knockout or *Hjv* knockout and *Slc39a14* (ZIP14) knockout failed to accumulate iron in the hepatocytes [41,42]. Extraction of NTBI by mice liver in a single pass is extremely high (58–76%) and independent of the amount of iron present in the liver [43,44,45]. So, we hypothesize that the majority of NTBI produced in the portal system (from orally administered iron) is highly efficiently taken up in the liver during the first passage through this organ and that this extraction by hepatocytes depends on ZIP14 [35]. Our data quantifies the extent of the hepatic first-pass NTBI extraction in human under various degrees of iron load mediated by ZIP14 (Figure 4).

This hypothesis corresponds with differences in organ distribution of iron in transfusion-dependent and transfusion-independent iron-overload [46,47,48,49]. NTBI released from the reticulo-endothelial system of the spleen might be extracted by the liver, whereas NTBI generated or infused in the systemic circulation will lead to iron influx and accumulation in other organs [50].

We observed that in non-transfusion-dependent hereditary anemia patients less iron from an oral or intravenous iron test dose was utilized for RBC production compared to hereditary hemochromatosis patients, independent from the extent of iron-overload. Iron utilization for red cell production ameliorated in hereditary spherocytosis patients after splenectomy. Presence of ineffective erythropoiesis or extravascular hemolysis results in less RBCs reaching or persisting in the circulation and consequently less iron in RBCs after 14 days. Recombinant transferrin injections in thalassemic mice improved iron availability for erythropoiesis, thereby underlining that despite systemic iron-overload the actual amount of iron available for erythropoiesis does not meet its demands [51]. So, in non-transfusion-dependent hereditary anemia patients, increased RBC turnover and pathologic iron trafficking result in low utilization of iron, which is improved in spherocytosis patients after splenectomy.

We showed that anemia and hemolysis are important determinants of time to disappearance of iron from the circulation. In non-transfusion-dependent hereditary anemia and iron deficiency anemia half-life of iron was reduced to half of hereditary hemochromatosis patients. Iron requirements of an active bone marrow are high. Iron acquisition in precursor RBCs depends on endocytosis of monoferric and diferric transferrin via TfR1 [52,53]. (Relative) iron deficiency will result in expansion of erythroid progenitors and increases in the amount of TfR1 per cell [54].

With exceptions, polymeric iron formulations have a low potential for LPI release; polymeric iron particles remain stable in the circulation until endocytosis and iron processing by macrophages [55]. Disappearance half-life of our test dose of transferrin-bound iron will not be comparable with disappearance half-life of polymeric iron formulations with high potential for LPI release, for example iron saccharate [55,56]. LPI in the circulation will load on transferrin, or enter endothelial cells in seconds [20], bind to stabilizing anti-oxidant ligands, enter intravascular buffering cells [57,58].

Determination of iron accumulation is crucial in diagnosing the occurrence and progression of many liver- and iron-related diseases. For interpretation of ferrokinetic data insight in the presence and abundancy of the various forms of iron in the circulation is of major importance. Distinct iron deposition profiles of liver zones in various models with iron homeostasis disorders were studied [59]. Uneven iron distribution was seen in livers of patients with hereditary hemochromatosis and in mice with hemochromatosis (*Hfe* knockout), showing the region with the highest iron concentration near the entrance site of the portal vein and hepatic artery. This is the pattern that can be expected in subjects who have a long term of increased liver iron retention due to iron influx through ferroportin that outnumbers the binding capacity of transferrin in the portal circulation.

In conclusion, insight in the composition of iron in the circulation (transferrin-bound, LPI/NTBI), pre-existing iron load and iron requirements are essential to explain human ferrokinetics. Our data supports that non-transfusion-dependent hereditary anemia patients have increased dietary iron absorption and decreased iron utilization despite fast uptake of transferrin-bound iron. We observed that all these characteristics are dramatically improved after splenectomy in patients with hereditary spherocytosis. Our data points towards the existence of significant hepatic scavenging of NTBI. We hypothesize on an important role for albumin as scavenger of NTBI, not only under iron-overloaded conditions, but as a safeguard for all conditions where the influx of Fe(II) via ferroportin outnumbers the iron binding capacity of plasma transferrin. We suggest that ZIP14 is required to extract NTBI during the first-pass of the liver.

## 4. Materials and Methods

### 4.1. Test Subjects

Iron absorption and ferrokinetic studies were performed from 1972 until 1994 as part of routine clinical practice in patients with iron-related health problems by the Iron Expert Clinic at the University Medical Center Utrecht, Utrecht, the Netherlands.

### 4.2. Patient Selection

Patients were retrospectively selected based on diagnosis (non-transfusion-dependent hereditary anemia, hereditary hemochromatosis and iron deficiency). Patients receiving iron reducing treatment, either deferoxamine or phlebotomies, were analyzed as separate groups. There was no iron reducing therapy during the two-week periods of examination of iron absorption and ferrokinetics. If more than one iron absorption and ferrokinetic study was performed all analyses were included in the final database. In the hereditary hemochromatosis group one patient was included with three analyses, in the non-transfusion-dependent hereditary anemia group two patients were included with two analyses and in the non-transfusion-dependent hereditary anemia group with iron reducing treatment and iron deficiency group one patient was included with two analyses.

Iron deficient patients and healthy controls were included as reference groups. Patients with iron deficiency due to iron absorption disorders were excluded, defined as iron uptake less than 50% and clinical suspicion of iron uptake disorder. The group of healthy controls was earlier described by Marx [60,61]. Healthy controls did not receive the intravenous iron test dose.

### 4.3. Iron Test Doses

In all subjects, first iron absorption was studied using a whole-body counter (WBC) as described by Marx [60,61,62]. In brief, a test dose of 7 mg ferrous ammonium sulphate, containing 1 mg Fe(II), labeled with 5 µCi ^59^Fe and 10 mg ascorbic acid (to prevent oxidation of iron in solution) was administered. The dose was ingested in the early morning after ten hours fasting; fasting was continued for two hours after ingestion. ^51^Chromium was added (as CrCl_3_, 40 µCi) to the oral test dose as non-absorbable indicator, for estimation of mucosal iron uptake. Red blood cell iron utilization (RBCIU) after the oral iron dose was measured 14 days after ingestion, after the last measurement of total body ^59^Fe and ^51^Cr.

The intravenous iron test dose was injected after overnight fasting. The dose contained approximately 10 µCi ^59^Fe bound to 5 mL autologous serum. Autologous serum was incubated with ^59^Fe for 30 min. Before injection, unbound iron was removed by the method described by Cavill [63]. In short, an anion exchange column (Amberlite-IRA 400-cl) removed all unbound ferric-citrate. Therefore, all ^59^Fe in the intravenous test dose was transferrin-bound iron. Before the injection of the intravenous test dose, background ^59^Fe in RBCs, due to RBCIU from the oral ^59^Fe administered at least 14 days earlier, was measured.

### 4.4. Measurement of Radioactivity

Radioactivity was measured with a whole-body counter, first at the Physical Laboratory, National Institute of Public Health, Bilthoven, The Netherlands, later (after moving the facility) at the University Medical Center Utrecht, Utrecht, The Netherlands. ^59^Fe activity was measured under the photopeak (1.00–1.40 MeV) and in the Compton area (0.40–1.00 MeV). ^51^Cr activity was measured under the photopeak (0.28–0.36 MeV) after subtraction of the ^59^Fe Compton effect. The first measurement was performed one hour after ingestion of the oral test dose; results were considered as 100%. ^59^Fe radioactivity of peripheral blood samples was measured with an automatic gamma counter and compared to standard solutions containing a preset amount of ^59^Fe. All measured values were corrected for background radiation and radioactive decay.

### 4.5. Iron Absorption Studies

The whole process of iron absorption includes mucosal iron uptake, mucosal transfer and retention. Absorption studies were performed according to the protocol described by Marx [60].

Mucosal iron uptake (the percentage of iron taken up by the mucosal cells from the lumen of the gut) The mucosal iron uptake was calculated from the amount of ^59^Fe and ^51^Cr within the body 24 hours after the test dose. If there had been no defecation, counting was postponed for 24 hours. ^59^Fe and ^51^Cr measurements at 24 (or 48) hours were expressed as percentage of the amount administered.

Mucosal uptake (%) = 100 × (^59^Fe − ^51^Cr)/(100 − ^51^Cr) (1)

Iron retention (the percentage of iron present in the body 14 days after ingestion)

Iron retention was calculated from the amount of iron in the body 14 days after the test dose as determined with a whole-body counter. ^59^Fe and ^51^Cr measurements at 14 days were expressed as percentage of the amount administered.

Iron retention (%) = 100 × (^59^Fe − ^51^Cr)/(100 − ^51^Cr) (2)

Mucosal iron transfer (the fraction of iron taken up by the mucosal cells at day one that is retained in the body).

Mucosal iron transfer (fraction) = iron retention (%)/mucosal iron uptake (%)(3)

### 4.6. Ferrokinetic Studies

Ferrokinetic methods used in the present study, based on early work by Finch and coworkers (1970), were described in detail before [64,65].

#### 4.6.1. Disappearance half-life of iron

The velocity of iron disappearance from the circulation was calculated after injection of intravenous iron, labeled with ^59^Fe. Half-life of iron was calculated from blood samples drawn at 0, 5, 15, 30, 45, 60, 90 and 120 min after injection based on the slope of the natural logarithm of radioactive decay.

#### 4.6.2. Iron incorporation in RBCs

The method for determining iron incorporation in RBCs was earlier described by Cook and others [25,61,64,65]. Incorporation of oral or intravenous administered iron was calculated from peripheral blood samples collected 14 days after iron administration. RBC iron utilization was expressed as percentage of the total amount of iron retained after an oral test dose or present after an intravenous test dose.

(4) RBCIU%=F 59e in 1 mL blood × blood volume mLtotal amount of F 59e in circulation × 100%

An oral test dose was given of 1 mg Fe(II) (as ferrous ammonium sulphate) labelled with 5 µCi ^59^Fe. The ^59^Fe-absorption was measured 14 days after ingestion of the test dose using a whole-body counter. The amount of ^59^Fe that reached the blood was calculated from the amount of ^59^Fe in the oral test dose and from the ^59^Fe absorbed:

(5) F 59e dose =F 59e absorption %100×F 59e administered orally cpm

This ^59^Fe dose is quantitatively comparable with an iron test dose injected directly into the portal vein. RBCIU after ^59^Fe injection was calculated by:

(6) F 59e RBCIU %=F 59e in 1 mL blood cpm × blood volume mLF 59e dose cpm×100%

#### 4.6.3. Liver iron retention

Intestinal absorbed iron enters the portal circulation and passes the liver before reaching the systemic circulation. Intravenous iron is injected directly into the systemic circulation. Liver iron retention (LIR) was defined as the difference in RBC iron incorporation from an oral and from an intravenous test dose, expressed as percentage of the RBC iron incorporation from an intravenous test dose.

(7) $$\mathrm{LIR}\left(\%\right)=\frac{\mathrm{RBCIU}\;\mathrm{intravenous}-\mathrm{RBCIU}\;\mathrm{oral}\;}{\mathrm{RBCIU}\;\mathrm{intravenous}}\times 100\%$$

### 4.7. Laboratory Assays

Red cell indices, hemolytic severity parameters and iron indices were measured with commercial assays used in clinical practice by the University Medical Centre Utrecht on the morning of ingestion of the oral iron test dose.

### 4.8. Statistical Analysis

Differences in means between different patient groups were tested with a one-way ANOVA, in case of significant differences a Tukey’s HSD test was performed testing all possible pairwise comparisons. Correlation analyses were reported using Pearson’s correlation coefficient. In order to correct for potential bias *bootstrapping* was performed to confirm significance [66]. Statistical significance was set at a two-sided *p* < 0.05. All calculations were performed with IBM SPSS Statistics v. 25.

## Figures and Tables

**Table 1:** Presented values are medians with interquartile ranges (IQR). Iron uptake, retention and transfer were calculated from the amount of ^59^Fe and ^51^Cr in the body 24 h after the oral iron test dose (uptake) and 14 days after the oral iron test dose (retention). Mucosal iron transfer is the fraction of iron retained after uptake. HA NTD hereditary anemia non-transfusion dependent; HH hereditary hemochromatosis; ID iron deficiency; irt iron reducing therapy (either phlebotomies or deferoxamine). AST aspartate aminotransferase; Hb hemoglobin; LD lactate dehydrogenase.

**Parameter**	**HA NTD**	**HA NTD-irt**	**HH**	**HH-irt**	**ID**	**Normal**
No. of patients	29	11	79	42	47	70
Male, %	79	82	66	86	26	56
Age, years	29 (22–37)	26 (15–39)	44 (35–56)	44 (37–54)	38 (23–47)	67 (26–70)
Laboratory parameters						
Hb, g/dL	10.5 (10.5–12.4)	12.4 (9.5–15.5)	15.5 (14.8–16.4)	16.0 (14.6–16.8)	11.1 (10.2–12.6)	15.5 (14.6–16.3)
Reticulocytes, × 10^9^/L	15 (12–39)	8 (5–243)	11 (7–17)	13 (7–26)	12 (8–15)	10 (6–15)
Ferritin, μg/L	407 (215–1800)	222 (109–367)	953 (440–1350)	70 (36–170)	7 (5–9)	NA
Iron saturation, fraction	0.74 (0.50–0.87)	0.76 (0.53–0.97)	0.85 (0.78–0.92)	0.69 (0.44–0.90)	0.08 (0.05–0.15)	0.36 (0.29–0.44)
Serum iron, μmol/L	33 (27–41)	35 (30–38)	38 (33–43)	34 (22–41)	7 (4–10)	21 (18–26)
AST, U/L	22 (16–42)	18 (13–27)	31 (20–44)	22 (15–26)	17 (15–23)	NA
LD, U/L	350 (277–644)	395 (311–593)	400 (325–473)	416 (323–459)	408 (372–455)	NA
Bilirubin, mg/dL	18 (8–44)	9 (7–28)	10 (8–16)	12 (9–18)	6 (4–9)	NA
Iron absorption studies						
Mucosal iron uptake, %	60 (54–77)	78 (55–87)	55 (47–72)	88 (81–93)	88 (75–96)	46 (29–55)
Mucosal iron transfer, fraction	0.76 (0.63–0.91)	0.90 (0.80–0.96)	0.84 (0.71–0.90)	0.97 (0.94–0.98)	0.96 (0.91–0.98)	0.62 (0.53–0.78)
Iron retention, %	42 (33–63)	66 (55–74)	45 (33–63)	85 (73–91)	81 (69–91)	25 (17–36)

## Abbreviations

DMT1	Divalent metal transporter 1
HH	Hereditary haemochromatosis
LIR	Liver iron retention
LPI	Labile plasma iron
NTBI	Non-transferrin-bound iron
RBC	Red blood cell
RBCIU	Red blood cell iron utilization
TfR1	Transferrin receptor 1
TSAT	Transferrin saturation
